# Commentary on: Aggadi N, Zeller K, Busey T. Quantifying the strength of firearms comparisons based on error rate studies. J Forensic Sci. 2024;70(1):84–97. https://doi.org/10.1111/1556‐4029.15646; Warren EC, Handley JC, Sheets HD. Cross entropy and log likelihood ratio cost as performance measures for multi‐conclusion categorical outcomes scales. J Forensic Sci. 2024;70(2):589–606. https://doi.org/10.1111/1556‐4029.15686

**DOI:** 10.1111/1556-4029.70165

**Published:** 2025-09-12

**Authors:** Geoffrey Stewart Morrison

**Affiliations:** ^1^ Forensic Data Science Laboratory Aston Institute for Forensic Linguistics, Aston University Birmingham UK; ^2^ Forensic Evaluation Ltd Birmingham UK

Editor,

Aggadi et al. [1] and Warren et al. [2] proposed methods for calculating likelihood ratios based on examiners' subjectively assigned categorical conclusions. Examiners participated in “black‐box studies,” and in response to each set of a questioned‐source item and one or more known‐source items (hereinafter “a test trial”), each examiner selected a categorical response from an ordinal scale such as the Association of Firearm and Tool Mark Examiners (AFTE). Range of Conclusions: “Identification,” “Inconclusive A,” “Inconclusive B,” “Inconclusive C,” “Elimination.”* The response data were then used to train a statistical model that could potentially be used to convert a categorical conclusion into a likelihood ratio, for example, the probability that an examiner would conclude “Identification” if the compared items came from the same source divided by the probability that the examiner would conclude “Identification” if the compared items came from different sources (and mutatis mutandis for other categorical conclusions such as “Inconclusive A,” “Inconclusive B,” “Inconclusive C,” and “Elimination”). The method described in Warren et al. [2] directly calculates Bayes factors (the Bayesian analogue of likelihood ratios) based on Dirichlet priors and raw count data for each response category. Response data are pooled across test trials and across examiners. The method described in Aggadi et al. [1] is more complex: Using response data pooled across examiners, an ordered probit model is fitted to the data from each test trial, then the models are averaged across test trials. This creates a latent dimension, and Bayes factors are calculated on the latent dimension. Using the Warren et al. [2] method, the likelihood‐ratio value corresponding to each categorical conclusion is directly calculated, and, in casework, the relevant likelihood‐ratio value would be substituted for (or provided in addition to) an examiner's categorical conclusion. Using the Aggadi et al. [1] method, a direct substitution is not possible. Aggadi et al. [1] proposed that six benchmark sets of items be visually displayed, placed according to their location on the latent dimension, and that the examiner use these as a guide to assign the location on the latent dimension for the set of items from the case. The likelihood‐ratio value corresponding to that location would then be used. Aggadi et al. [1] did not demonstrate implementation of this part of its method. Aggadi et al. [1] demonstrated application of the earlier parts of its method to firearms data (Busey & Coon [3] had earlier demonstrated application to fingerprint data), and Warren et al. [2] demonstrated application of its method to data from multiple fields, including fingerprints, firearms, bloodstain‐pattern analysis, footwear, and handwriting.

The likelihood‐ratio framework is the logically correct framework for interpretation of forensic evidence, and its use is advocated by key organizations [4, 5, 6, 7, 8, 9]. The promotion in Aggadi et al. [1] and Warren et al. [2] (and Busey & Coon [3]) of the likelihood‐ratio framework in fields such as firearms examination and friction‐ridge examination is, therefore, welcome.

Calculation of likelihood ratios using methods based on relevant data, quantitative measurements, and statistical models would be preferable to using methods based on human perception and subjective judgment because the former methods would be more transparent and more easily reproduced, they would be more resistant to cognitive bias, and they would be more easily calibrated and validated under casework conditions [10]. In research applied to firearms examination and friction‐ridge examination, there has been progress on methods for calculating likelihood ratios based on relevant data, quantitative measurements, and statistical models, for example, [11, 12, 13, 14, 15, 16], but this has not yet led to widespread adoption of such methods in casework. Methods that, instead, convert to likelihood ratios the subjectively assigned categorical conclusions that examiners make in their existing practice could be a faster route to the adoption of the likelihood‐ratio framework in these fields, and it could be a stepping stone toward acceptance in those fields of methods that calculate likelihood ratios based on relevant data, quantitative measurements, and statistical models [17, 18].

Unfortunately, the methods presented in Aggadi et al. [1] and Warren et al. [2] are not appropriate for calculating a likelihood ratio that is meaningful in the context of a case. The reasons for this, and outlines of potential solutions, are discussed below.

In order to provide a meaningful likelihood ratio in the context of a case, the response data used to train the statistical model would have to be representative of the performance of the particular examiner who performed the forensic comparison for that case (or, if the process for determining a categorical response, e.g., the ACE‐V process, involved two examiners, the response data used to train the statistical model would have to be representative of the performance of the particular pair of examiners who performed the forensic comparison for that case). The particular examiner could perform substantially better or substantially worse than the average of multiple examiners, in which case a model trained on pooled data from multiple examiners would not be representative of the performance of the particular examiner. Thus, for example, the probability that the particular examiner would conclude “Identification” if the compared items came from the same source divided by the probability that the particular examiner would conclude “Identification” if the compared items came from different sources could be quite different from the likelihood ratio calculated by a model trained on pooled data from multiple examiners. The models in both Aggadi et al. [1] and Warren et al. [2] were trained using pooled data from multiple examiners; thus, they could not be used to calculate a likelihood ratio that would be appropriate for any particular examiner to present to a court in any particular case.

An obstacle to training a model based on data from a particular examiner would be the difficulty of collecting sufficient response data from that practitioner. The examiner would have to provide responses to a large number of same‐source test trials and to a large number of different‐source test trials. Morrison [17] proposed a Bayesian method to reduce this difficulty and, as a proof of concept, demonstrated it using beta‐binomial models. In that method, a (relatively) large amount of response data from multiple examiners (excluding the particular examiner) is used to train a same‐source prior model and to train a different‐source prior model. These models provide informed priors for the performance of a particular examiner. The smaller amount of response data available from the particular examiner is used to update the prior models to posterior models, and a Bayes factor is calculated using the expected values of the same‐source and different‐source posterior models. The particular examiner would not have to provide responses to large numbers of test trials up front. Blind test trials [19, 20] could be inserted into the examiner's workflow, and as the amount of available response data increased over time, the models could be updated. Initially, when only a small amount of response data from the particular practitioner would be available, the likelihood ratios calculated would be more influenced by the average performance of multiple examiners, but, over time, as the amount of available response data from the particular practitioner increased, the likelihood ratios calculated would be more reflective of the performance of that examiner.

In order to provide a meaningful likelihood ratio in the context of a case, the response data used to train the statistical model would not only have to be representative of the performance of the particular examiner who performed the forensic comparison for that case, but they would also have to be representative of the performance of that examiner under conditions that reflect the conditions of that particular case. This would include that the items used in the test trials reflect the conditions of the questioned‐source and known‐source items in the case, for example, in comparison of a fingermark and a fingerprint, the number of minutiae visible in the fingermark and visual interference due to the type of surface on which the fingermark was deposited might be relevant conditions of the questioned‐source item, and the fingerprint being a controlled high‐quality digital capture might be the relevant condition of the known‐source item, and in comparison of fired cartridge cases, relevant conditions might include the caliber of the ammunition, the gross shape of the firing pin, and the number of cartridges fired from the known firearm. Determining what constitutes relevant sets of casework conditions and what set of conditions a particular case corresponds to would require subjective judgment and subject‐area expertise (in the same way as these are required for selecting relevant data for calibration and validation [21], in fact, conversion of an examiner's subjectively assigned categorical conclusion to a likelihood ratio could be considered calibration [22]).

Some sets of conditions will be more challenging, leading to poorer performance, and some sets of conditions will be less challenging, leading to better performance. More challenging conditions would be expected to result in more “Inconclusive” conclusions and, concomitantly, fewer “Identification” and “Exclusion” conclusions than would be the case for less challenging conditions. For a well‐calibrated likelihood‐ratio‐calculation system [23], more challenging conditions would result in likelihood ratios that tended to be closer to the neutral value of 1 than would be the case for less challenging conditions. For example, Weber et al. [24] Figure 8 shows Tippett plots of results from a calibrated forensic‐voice‐comparison system—the shorter the duration of the speech in the questioned‐speaker recording, the more challenging the condition, and the closer the log likelihood ratios tend to be to the neutral log‐likelihood‐ratio value of 0. Likelihood ratios calculated under one set of casework conditions could, therefore, be substantially different from likelihood ratios calculated under a different set of casework conditions. Calculating likelihood ratios from models trained on pooled data from multiple different conditions could be substantially different from what the likelihood‐ratio values would be if the model were trained on data that reflected the conditions of a particular case. It was not always clear whether a model in Aggadi et al. [1] and Warren et al. [2] was trained on response data from test trials that were all intended to reflect a single set of casework conditions or whether a model was trained on response data pooled from test trials that reflected a variety of different sets of casework conditions. Warren et al. [2] included four models, each trained and tested on one of four different fired‐cartridge‐case datasets, each of which resulted in a different log‐likelihood‐ratio‐cost (*C*_llr_) value [25]. The different *C*_llr_ values could (at least in part) have been due to differences in how challenging the conditions of the test trials were.

In order to train a model that calculates a meaningful likelihood ratio in the context of a particular case, the particular examiner who performed the forensic comparison for that case would have to provide responses to a large number of same‐source test trials and to a large number of different‐source test trials in which the conditions of the items in each test trial reflected those of the items in that case. In order to be able to calculate likelihood ratios in each of multiple different sets of casework conditions, the examiner would have to provide responses to a large number of test trials in each of the multiple different sets of conditions. This would further increase the difficulty of data collection. To reduce this difficulty, a statistical model could be developed that includes terms that encode how challenging the conditions are. Subject‐area expertise could be used to define categories of relevant sets of casework conditions, and the model would include terms related to those categories. Similarly, terms could be included to encode how well each examiner performs on average, and there could also be examiner‐by‐condition interaction terms. Interaction terms would account for situations in which, for example, two examiners performed equally well on one condition, but one examiner performed better than the other on another condition. A suitable model could be logistic regression, the output of which could be manipulated to calculate a likelihood ratio for each combination of examiner, condition category, and categorical response. If the amount of available response data for a particular examiner in a particular condition category were limited (or even non‐existent), the likelihood‐ratio values for that examiner in that condition category would then be partially (or wholly) interpolated or extrapolated from that examiner's performance under other condition categories and from other examiners' performance under that condition category.

The data used to train models in Aggadi et al. [1] and Warren et al. [2] came from black‐box studies. Cuellar et al. [26] criticized the designs of existing firearms black‐box studies on multiple grounds, including on the ground that the examiners knew they were being tested and, because of this, may have behaved differently compared to when they perform casework. In fact, Scurich et al. [20] found substantially higher rates of “Inconclusive” responses when firearms examiners realized that blind test trials inserted into their workflow were tests, compared to when they believed that the test trials were real casework. In order to calculate meaningful likelihood ratios, response data used for training statistical models would have to reflect casework conditions. Because of differences in performance when examiners know that they are being tested versus when they do not know they are being tested, such data would have to be collected using blind test trials (and responses would have to be excluded from the training data in instances in which an examiner realized that a blind test trial was a test [20]).

Methods such as those in Aggadi et al. [1] and Warren et al. [2] that convert examiner's subjectively assigned categorical conclusions into likelihood ratios could promote the adoption of the likelihood‐ratio framework in fields such as friction‐ridge examination and firearms examination; however, as outlined above, there are a number of refinements that would have to be made to the statistical models and the training data before such methods could be used by particular examiners to provide meaningful likelihood ratios for comparisons of particular sets of items in particular cases.

## CONFLICT OF INTEREST STATEMENT

The author declares that they have no known competing financial interests or personal relationships that could have appeared to influence the work reported in this paper.

## DISCLAIMER

Unless explicitly otherwise attributed, all opinions expressed in the present paper are those of the author, and they should not be construed as representing policies or positions of any organizations with which the author is associated.

## Footnotes

*
https://temp.afte.org/about‐us/what‐is‐afte/afte‐range‐of‐conclusions.
